# Identifying relevant intersections in relation to motivation and attempt to stop smoking by using a combination of methods to develop robust predictive models and resampling techniques: A cross‐sectional study of the German population

**DOI:** 10.1111/add.70045

**Published:** 2025-03-21

**Authors:** Sabina Ulbricht, Adrian Richter, Daniel Kotz, Sabrina Kastaun

**Affiliations:** ^1^ Department SHIP‐KEF, Institute for Community Medicine University Medicine Greifswald Greifswald Germany; ^2^ Epidemiology and Health Services Research, German Rheumatology Research Centre Berlin Berlin Germany; ^3^ Institute of General Practice, Addiction Research and Clinical Epidemiology Unit, Centre for Health and Society, Medical Faculty and University Hospital Düsseldorf Heinrich Heine University Düsseldorf Germany; ^4^ Department of Behavioural Science and Health University College London London UK; ^5^ Institute of General Practice, Patient‐Physician Communication Research Unit, Centre for Health and Society, Medical Faculty and University Hospital Düsseldorf Heinrich Heine University Düsseldorf Germany

**Keywords:** cross‐sectional population survey, interaction, intersectionality, motivation to stop, quit attempt, smoking

## Abstract

**Aims:**

To illustrate robust intersections of co‐occurring factors for two predictors of smoking cessation, motivation to stop smoking (MTSS) and past year‐quit attempts (QA), by using means to develop robust predictive models such as bootstrap resampling, scoring rules to evaluate the predictive accuracy and spline functions.

**Design, Setting and Participants:**

Cross‐sectional data from the German Study on Tobacco Use (DEBRA). Past‐years smokers (≥18 years, *n* = 13 245) from 22 survey waves (2016–2020) were included. The sample (mean age 46.8 years, 46.7% women) was randomly divided into learning (70%) and validation data (30%). Less than 20% in both data sets had tried to stop smoking within the preceding 12 months.

**Measurements:**

Multinomial regression (for MTSS) and logistic regression (for QA) were used to evaluate whether age, sex, education, monthly net household income per person and the region of residence form intersections with relevant differences in the two outcomes.

**Findings:**

MTSS compared with the absence of MTSS was associated with middle [95% confidence interval (CI) = 1.02–1.39] and high education (95% CI = 1.37–1.98). Regarding MTSS, the highest probabilities were observed in participants aged 30 to 50 years from lower and middle (30–40 years) income groups. Regarding QA, the probability of at least one past‐year QA was highest in females aged between 20 and 40 years and independent from educational level. Similar probabilities in males were seen only among those from the highest educated group. The predictive accuracy of the results was reduced by 3.1% for MTSS and 3.4% for QA when comparing learning with validation data.

**Conclusions:**

This German study provides compelling evidence linking highest motivation to stop smoking to those aged 30 to 50 years with lower or middle household income. Regardless of educational level, females' probabilities of reporting at least one past‐year quit attempt appears to be highest in those aged 20 to 40 years. These findings highlight the need for adopting an intersectional approach when studying predictors of smoking cessation.

## INTRODUCTION

Tobacco use causes 8 million premature deaths worldwide each year, predominantly from respiratory diseases, cardiovascular diseases and cancer diseases [1]. Although there was a decline in tobacco use over the past decades in high income countries, including Germany [2], socio‐economic inequalities in smoking prevalence have persisted or increased over time [3, 4, 5, 6, 7].

Smoking prevalence is determined by uptake of smoking and smoking cessation. Socio‐demographic factors, which are known to impact both determinants comprise sex and education [8, 9]. Motivational factors, including the intention or desire to stop smoking have been identified as important predictors of quit attempts [10] and as a consequence smoking cessation.

Although the majority of studies considered attributes such as age, sex or education as mutually independent factors, a more recent study [11] has examined the potentially synergistic effects of such characteristics on smoking cessation using the intersectionality framework [12, 13]. In line with this framework, smoking cessation outcomes may be shaped by the interaction of several attributes of a person. This interplay can result in different levels of motivation to stop smoking (MTSS) and may also manifest itself in different probabilities of quit attempts. For example, a younger female who lives in an urban region and has an above‐average level of education may be more motivated to stop smoking than an older female who lives in a rural region and has a lower educational level [12, 13]. Recognising these multiple intersecting dimensions is important to understand how age and sex interact with other attributes (e.g. region of residence) in order ‘to improve the understanding of how multiple factors relate to health’ [11]. Unfortunately, the approach proposed in this study [11] is known to generate spurious effects because of categorisation of continuous covariates (e.g. age) [14] and because of multiple testing in too complex regression models [15]. In consequence, some of the intersections may only appear to be different with regard to the outcome, but the result is likely to be irreproducible. Similar findings regarding the susceptibility for spurious effects were made for the application of multilevel models in the context of intersectionality research [16].

Generalized additive models (GAM) [17] can be used to allow for flexible modelling and the inclusion of interactions while avoiding categorisation of continuous covariates. The built‐in algorithm of GAMs selects the optimal complexity of functional forms. However, there is no inherent mechanism to control for the complexity in terms of the number of interaction terms. To enhance robustness and generalisability of results in studies investigating complex questions of intersectionality we propose a combination of established methods for developing predictive models [18].

To illustrate robust intersections of co‐occurring factors (age, sex, education, monthly net household income and the region of participants' residence) for two smoking cessation outcomes: MTSS (current smokers) and past year‐quit attempts (QA, among past‐year smokers, including current smokers and ex‐smokers who had stopped smoking within the preceding 12 months) this study uses a three‐step process. First, we divided the large corpus of the German Study on Tobacco Use (DEBRA: ‘Deutsche Befragung zum Rauchverhalten’) into learning and validation data. Second, using bootstrap resampling, we evaluated different regression models comprising interactions between socio‐demographic factors and varying functional forms of continuous covariates to examine their association with both outcome measures. Third, the best model for both outcomes was examined for robustness in validation data.

## MATERIAL AND METHODS

### Data source

The ongoing DEBRA study is a representative, nationwide household survey on the use of tobacco and nicotine products (https://www.debra-study.info/). The protocol of the general study design has been published [19]. A registration of the study can be found at German Clinical Trials Register (registration numbers DRKS00011322, DRKS00017157 and DRKS00028054). Data from computer‐assisted face‐to‐face household interviews are collected every other month from individuals age ≥14 years between June/July 2016 and April/July 2023. The article presents data of 22 survey waves (2016–2020) with a total of 13 245 past‐year smokers, including 12 784 current smokers and 461 ex‐smokers who had stopped smoking within the preceding 12 months. A detailed study protocol was pre‐registered on the Open Science Framework (https://osf.io/ub3w7).

## MEASURES

### Outcome variables

The first outcome examined with regard to intersections was the level of MTSS, measured among current smokers and by the German version of the Motivation to Stop Scale (MTSS) [20, 21]. MTSS is rated on a 7‐level scale, ranging from lowest level 1 (I do not want to stop smoking) to highest level 7 (I really want to stop smoking and intend to in the next month).

The second outcome considered was past‐year QA. Past‐years smokers were asked: ‘How many serious attempts to stop smoking have you made in the last 12 months? By serious attempt I mean you decided that you would try to make sure you never smoked again. Please include any attempt that you are currently making and please include any successful attempt made within the last year’.

### Intersectionality measures

This study is interested in possible intersections formed by the covariates: age, sex, educational level, monthly net household income and region of residence. Age and monthly net household income are considered as continuous variables in the models. Monthly net household income was calculated per person/month on the household (for more details https://osf.io/387fg/) and coded from 0 (€0 income) to 7 (>€7000), according to an equalisation technique of the Organisation for Economic Cooperation and Development [22]. Sex (female or male), educational level (low = junior high school equivalent or no graduation as reference vs. middle = secondary school equivalent or high = high school equivalent or advanced technical college equivalent) and participant's region of residence (metropolitan, urban, vs. rural as reference) were evaluated as categorical variables. In line with a previous German cohort study [23], participant's region of residence was assessed using the administrative municipality district size that was collapsed into three categories: >500 000 = metropolitan area, 20 000 to 500 000 = urban area and <20 000 = rural area.

### Potential confounding measures

Strength of urges to smoke was assessed by using the second item of the German version of the Strength of Urges to Smoke Scale [24]. The item consists of a simple rating of strength of urges on a normal smoking day ranging from 0 (none) to 5 (extremely strong) [25].

## Statistical analysis

### Initial data analysis

We first examined longitudinal changes across survey waves from 2016 and 2023 with regard to all intersectionality measures (please see above), the prevalence of smoking, MTSS and the number of QAs (Figures S1–S8). The data indicated a considerable increase in the prevalence of smokers during the coronavirus disease 2019 pandemic [26, 27] (Figure S6). Therefore, we suspected an influence of the pandemic on the study outcomes, which has been described in the study protocol. Subsequently, the final sample was restricted to those being surveyed between 2016 and 2020 (waves 1–22; *n* = 13 245 past‐year smokers). We deviated from our assumptions in the protocol by including the covariate time in all models, because considerable time trends were found in earlier waves of the DEBRA study with regard to the outcome of at least one QA (Figure S7).

### Analysis of intersectionality

Statistically, the explorative investigation of intersectionality entails the consideration of multiple interaction terms in regression models, which markedly increases model complexity. It is well established that overly complex models are susceptible to overfitting the data [28] (i.e. a too complex model fits also to the noise in the data). With regard to intersectionality research, there is a significant risk of false‐positive results and overfitting if multiple interaction terms are examined simultaneously [29]. Resampling methods, such as cross‐validation or bootstrap, are computationally demanding but can assist in avoiding overfitting. In essence, resampling enables the identification of a model that may not be optimal for a single data set but is, nevertheless, the most accurate on average across multiple randomly generated data representations.

Categorization of continuous covariates, which has been discouraged since decades [28, 30, 31], increases model complexity further. The perceived benefits of categorization are, among others, eased model interpretation and handling of non‐linearity. However, it has been shown that this approach generates biased estimates and is statistically inferior to the use of spline functions in case of non‐linearity. To overcome interpretability issues when using spline functions several best‐practice approaches are available [32].

**FIGURE 1:**
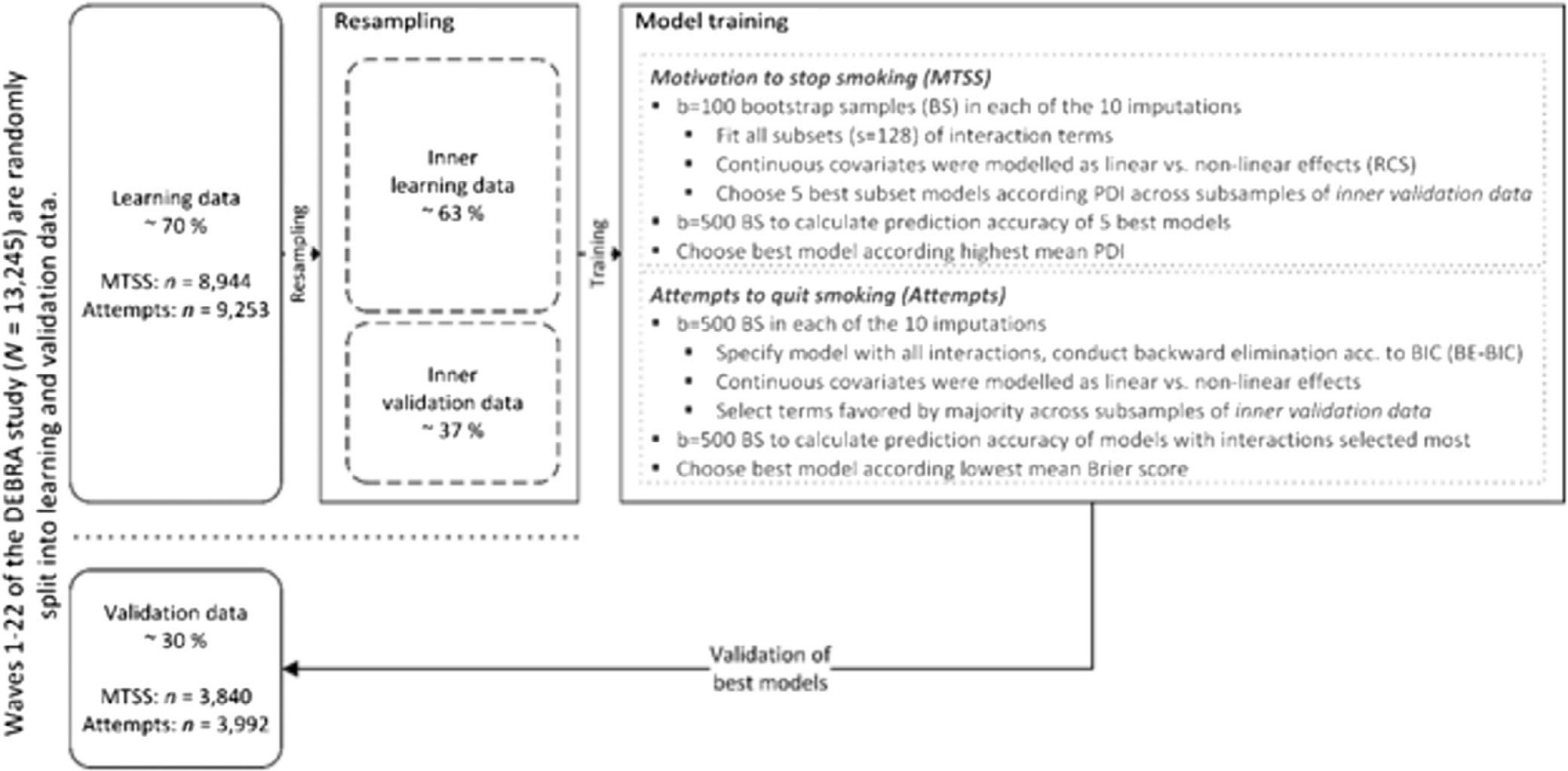
Scheme of model training and validation algorithm.

The approach adopted in this study is based on a recent simulation study that has shown superiority of resampling‐based methods over heuristic approaches when examining multiple intersections [29]. The algorithm of this approach is described below (please see: Model training and validation, Figure 1).

## Regression models

### MTSS

For the first outcome, application of ordinal regression models was planned in the study protocol. Initial analysis revealed violations of the proportional odds assumption [33], and therefore, multinomial regression models were chosen (Table S1). The seven categories of the MTSS‐scale were collapsed into three categories. The first category (absence of motivation) represents individuals without any intention to quit smoking (MTSS‐scale level 1 ‘I don't want to stop smoking’). The second category (unspecific motivation) covers those with moderate desire, but no intention to quit smoking (MTSS‐scale level 2 and 3, e.g. ‘I think I should stop smoking but don't really want to’) [21]. Individuals who reported a strong desire, but no intention to quit or a moderate or strong desire and an intention to quit (MTSS‐scale level 4, 5, 6 and 7, e.g. ‘= I really want to stop smoking and intend to in the next months.’) were assigned to category three (motivation). The approach of collapsing categories is only partially methodologically justified by the violation of the proportional odds assumption. Nevertheless, the categories were collapsed on the basis of content‐related decisions and are not methodologically supported. As most variable selection approaches are not designed for multinomial regression models [34], best subset selection has been chosen [35] (please see: Model training and validation).

### QA

Our second outcome, the number of QAs, was recoded into a dichotomous variable ‘past‐year quit attempt’ (yes, ≥1 attempt vs. none). Multiple logistic regression models were used to describe the odds within the last 12 months. We followed a 2‐step approach for the choice of covariates: the functional form and interaction term [36, 37, 38] were chosen using backward elimination (BE) in combination with Bayesian information criteria (BIC) [39] (please see: Model training and validation).

### Model training and validation

Step 1: data of the 22 survey waves comprised 13 245 past‐year smokers, including 12 784 current smokers and 461 ex‐smokers who had stopped smoking within the preceding 12 months. The data were randomly divided into learning data (70%) and validation data (30%).
Step 2: within the learning data, repeated bootstrap samples are used to create inner learning and validation data. Different models are then fitted to inner learning data and predictions of outcomes are computed for inner validation data. The best model is selected according to best prediction accuracy, for instance, the highest polytomous discrimination index (PDI) [40, 41, 42] for the first outcome (MTSS) and lowest Brier score [43] for the second outcome (QA). As a final step, models comprising an interaction terms were tested against the same model without the interaction term using likelihood ratio tests.
Step 3: the predictive accuracy of the final model was evaluated in independent validation data (Figure 1).

### Missing data

The overall amount of missing data in the DEBRA study is very low and did not exceed 8% (Table 1). Nevertheless, missingness was found to be selective in subgroups of the data, for example, males with higher education more frequently denied information on smoking habits (Figure S9). We, therefore, decided to apply multiple imputation [44] using the R package mice [45]. The imputation model comprised all intersectionality measures and confounder of the analysis models. To avoid model optimism regarding the prediction accuracy, imputations were done separately for learning data (m = 10 imputations) and validation data (m = 20 imputations). Given the overall low number of missing values, we considered 10 imputations in the learning data to be sufficient and in alignment with recommendations [46].

**TABLE 1:** Characteristics of the study sample.^a^ ^,^ ^b^

	Learning data		Validation data	
	Past‐year smokers	Current smokers	Past‐year smokers	Current Smokers
*n*	9253	8944	3992	3840
Age (y)				
Mean (SD)	46.8 (16.7)	46.9 (16.7)	46.8 (16.9)	46.9 (16.9)
Median [Min, Max]	48.0 [18.0, 95.0]	48.0 [18.0, 95.0]	48.0 [18.0, 99.0]	48.0 [18.0, 99.0]
Sex (*n*, %)				
Male	4941 (53.4)	4778 (53.4)	2115 (53.0)	2034 (53.0)
Female	4312 (46.6)	4166 (46.6)	1877 (47.0)	1806 (47.0)
Education (*n*, %)				
Low	3114 (33.7)	3040 (34.0)	1286 (32.2)	1252 (32.6)
Middle	3623 (39.2)	3499 (39.1)	1641 (41.1)	1583 (41.2)
High	2391 (25.8)	2286 (25.6)	1011 (25.3)	953 (24.8)
Missing	125 (1.4)	119 (1.3)	54 (1.4)	52 (1.4)
Region of residence (*n*, %)				
Rural	3323 (35.9)	3202 (35.8)	1456 (36.5)	1405 (36.6)
Urban	3995 (43.2)	3869 (43.3)	1753 (43.9)	1676 (43.6)
Metropolitan	1935 (20.9)	1873 (20.9)	783 (19.6)	759 (19.8)
Net household income^c^				
Mean (SD)	1.47 (0.818)	1.47 (0.819)	1.47 (0.790)	1.47 (0.788)
Median [Min, Max]	1.39 [0, 7.00]	1.39 [0, 7.00]	1.43 [0, 7.00]	1.43 [0, 7.00]
Missing (*n*, %)	2 (0.0)	1 (0.0)	1 (0.0)	0 (0)
Cigarettes per day^d^				
Mean (SD)	13.8 (8.27)	13.8 (8.23)	14.0 (8.01)	14.0 (8.01)
Median [Min, Max]	14.0 [1.00, 70.0]	14.0 [1.00, 70.0]	14.0 [1.00, 80.0]	14.0 [1.00, 80.0]
Missing (*n*, %)	601 (6.5)	518 (5.8)	249 (6.2)	213 (5.5)
Heaviness of smoking index^d^				
Mean (SD)	4.07 (1.54)	4.07 (1.54)	4.09 (1.52)	4.09 (1.52)
Median [Min, Max]	4.00 [2.00, 8.00]	4.00 [2.00, 8.00]	4.00 [2.00, 8.00]	4.00 [2.00, 8.00]
Missing (*n*, %)	961 (10.4)	652 (7.3)	430 (10.8)	278 (7.2)
Urges to smoke (*n*, %)				
None	668 (7.2)	477 (5.3)	305 (7.6)	210 (5.5)
Light	2046 (22.1)	1991 (22.3)	863 (21.6)	839 (21.8)
Medium strong	3708 (40.1)	3664 (41.0)	1615 (40.5)	1596 (41.6)
Strong	2109 (22.8)	2100 (23.5)	886 (22.2)	879 (22.9)
Very strong	519 (5.6)	517 (5.8)	235 (5.9)	232 (6.0)
Extremely strong	92 (1.0)	92 (1.0)	42 (1.1)	42 (1.1)
Missing	111 (1.2)	103 (1.2)	46 (1.2)	42 (1.1)
Quit attempt (≥1 QA, last 12 months)^d^ (*n*, %)				
No	7141 (77.2)	7141 (79.8)	3067 (76.8)	3067 (79.9)
Yes	1689 (18.3)	1380 (15.4)	778 (19.5)	626 (16.3)
Missing	423 (4.6)	423 (4.7)	147 (3.7)	147 (3.8)
MTSS^e^				
1	4221 (45.6)	4221 (47.2)	1889 (47.3)	1889 (49.2)
2	2347 (25.4)	2347 (26.2)	979 (24.5)	979 (25.5)
3	961 (10.4)	961 (10.7)	348 (8.7)	348 (9.1)
4	307 (3.3)	307 (3.4)	145 (3.6)	145 (3.8)
5	786 (8.5)	786 (8.8)	312 (7.8)	312 (8.1)
6	100 (1.1)	100 (1.1)	55 (1.4)	55 (1.4)
7	86 (0.9)	86 (1.0)	46 (1.2)	46 (1.2)
Missing	445 (4.8)	136 (1.5)	218 (5.5)	66 (1.7)

Abbreviations: Max, maximum; Min, minimum; MTSS, motivation to stop smoking; QA, past year‐quit attempts.

^a^ The study sample comprised 13 245 past‐year smokers, including 12 784 current smokers and 461 recent ex‐smokers (i.e. individuals who had stopped smoking within the preceding 12 months).

^b^ The data set of past‐years smokers and current smokers were split into training data (70%) and validation data (30%).

^c^ Net household income was standardised into a value range of 0 = lowest to 7 = highest, according to an equalisation technique of the Organisation for Economic Cooperation and Development.

^d^ For the second outcome ‘attempts to quit smoking’ recent ex‐smokers are included. Therefore, learning data (validation data) for the second outcome comprised additional *n* = 309 (*n* = 152) participants.

^e^ Not assessed among recent ex‐smokers.

Final estimates of coefficients were obtained after fitting the best models to the learning data containing multiple imputations of missing data. Pooled estimates are presented as OR and respective 95% CI. Marginal means [47] were also calculated according Rubins' rule and illustrated using the R package *ggeffects* [48]. Finally, model estimates were used to predict both outcomes in the validation data.

### Computations

We used parallelized R code (R version 4.3.1, [49]; RStudio version 2023.06.1.) supported by the R packages *foreach* and *doParallel* [50] and a high performance cluster computer of the University of Greifswald [51]. All code used is publicly available in an open GitLab repository [52].

## RESULTS

### Characteristics of participants

The sample of past‐year smokers (*n* = 13 245) was composed in the learning data of 4312 women (46.6%) and 4941 men (53.4%) with a mean age of 46.8 years (SD = 16.7) (Table 1). The validation data included 1877 females (47.0%) and 2115 males (53.0%) with a mean age of 46.8 years (SD = 16.9). Among them, 45.6% (learning data) and 47.3% (validation data) reported the absence of MTSS. Less than 20% in both data sets had tried to stop smoking within the preceding 12 months (learning data = 18.3%, validation data = 19.5%).

### First outcome: MTSS

Table 2 shows that an unspecific motivation compared with the absence of MTSS was associated with female sex (OR = 1.18; 95% CI = 1.08–1.30), high versus middle education (OR = 1.13; 95% CI = 1.01–1.26), high versus low education (OR = 1.33; 95% CI = 1.16–1.52) and participant's residence in an urban versus rural area (OR = 1.12; 95% CI = 1.01–1.24). Further, a MTSS, compared with the absence of motivation, was associated with both middle (OR = 1.19; 95% CI = 1.02–1.39) and high education (OR = 1.65; 95% CI = 1.37–1.98), and participant's residence in an urban versus rural area (OR = 1.17; 95% CI = 1.01–1.35). When evaluating the interaction of age and net household income, changes in MTSS depend on variation in these variables. Therefore, the respective main effects of age and monthly net household income are not interpreted [28].

**TABLE 2:** Multinomial regression analyses of the MTSS.^a^

Term	MTSS: unspecific^a^		MTSS: higher^a^	
	OR	95% CI	OR	95% CI
(Intercept)	0.84	[0.47; 1.5]	0.65	[0.31; 1.38]
Strength of urges to smoke				
None	0.64	[0.51; 0.8]	0.63	[0.47; 0.86]
Light	0.75	[0.67; 0.85]	0.85	[0.72; 1]
Medium strong	1.00	Ref.	1.00	Ref.
Strong	0.86	[0.76; 0.97]	0.73	[0.62; 0.86]
Very strong	0.72	[0.59; 0.88]	0.43	[0.31; 0.61]
Extremely strong	0.52	[0.31; 0.85]	0.80	[0.44; 1.46]
Time^b^				
2016	1.00	Ref.	1.00	Ref.
2017 vs. 2016	1.26	[1.11; 1.44]	0.84	[0.71; 0.99]
2018 vs. 2016	1.20	[1.04; 1.38]	0.70	[0.58; 0.83]
2019 vs. 2016	1.15	[1.01; 1.30]	0.59	[0.50; 0.70]
2020 vs. 2016	1.25	[1.04; 1.50]	0.54	[0.42; 0.70]
Age^b^				
20	1.00	Ref.	1.00	Ref.
30 vs. 20	1.00	[0.81; 1.24]	2.04	[1.50; 2.78]
40 vs. 20	1.00	[0.80; 1.25]	1.88	[1.35; 2.63]
50 vs. 20	1.04	[0.85; 1.28]	1.55	[1.15; 2.09]
60 vs. 20	1.10	[0.89; 1.36]	1.87	[1.36; 2.58]
70 vs. 20	1.01	[0.81; 1.27]	1.32	[0.94; 1.85]
80 vs. 20	0.74	[0.55; 1.00]	0.50	[0.30; 0.84]
Sex				
Male	1.00	Ref.	1.00	Ref.
Female	1.18	[1.08; 1.3]	1.13	[1; 1.29]
Education				
Low	1.00	Ref.	1.00	Ref.
Middle	1.13	[1.01; 1.26]	1.19	[1.02; 1.39]
High	1.33	[1.16; 1.52]	1.65	[1.37; 1.98]
Region				
Rural	1.00	Ref.	1.00	Ref.
Urban	1.12	[1.01; 1.24]	1.17	[1.01; 1.35]
Metropolitan	0.92	[0.81; 1.05]	0.91	[0.76; 1.09]
Household income^b^ ^,^ ^c^ ^,^ ^d^				
[0–1]	1.00	Ref.	1.00	Ref.
[1–2] vs. [0–1]	1.10	[0.88; 1.38]	0.74	[0.55; 0.99]
[2–3] vs. [0–1)	1.00	[0.76; 1.31]	0.59	[0.41; 0.85]
[3–4] vs. [0–1]	0.79	[0.52; 1.18]	0.49	[0.27; 0.90]

Abbreviation: MTSS, motivation to stop smoking

^a^ Reference category: MTSS: absence of motivation.

^b^ Continuous covariates were modelled using restricted cubic splines. Respective model estimates were evaluated at specified values of the covariate and contrasted to a reference category; estimates and confidence intervals were calculated using Rubins' rule.

^c^ Net household income was standardised into a value range of 0 = lowest to 7 = highest, according to an equalisation technique of the Organisation for Economic Cooperation and Development [27].

^d^ Evaluation of the interaction effect is omitted in the table but explicitly shown in Figure 2.

**FIGURE 2:**
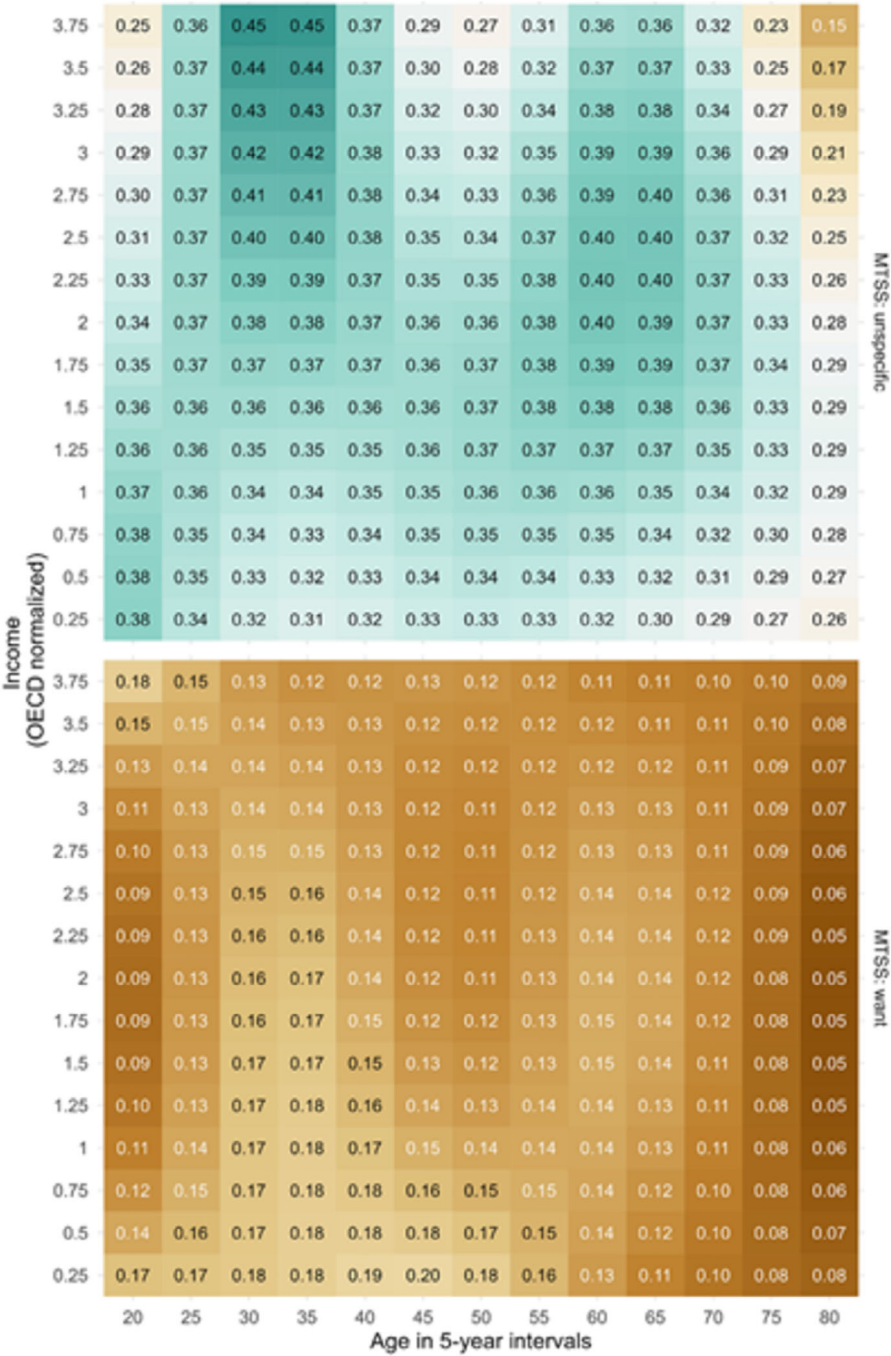
Probabilities of reporting an unspecific motivation or am motivation to stop smoking: interaction 633 of participants age (x‐axis) and monthly net household income (y‐axis).

Estimated marginal means for the probabilities of absence and MTSS, stratified by monthly net household income, varied non‐linearly over smokers' age from high net income groups versus low monthly net income groups (Figure 2). A MTSS was more likely in participants between 30 and 40 years from lower income groups compared to their counterparts with higher monthly net household income. As outlined in Figure 2, the highest probabilities of reporting a MTSS in participants between 40 and 50 years were seen among those from the lowest income groups. No other intersection with regard to MTSS was detected.

### Second outcome: QA

In the model that included QA as dependent variable, higher monthly net household income was associated with lower odds of reporting a past‐year QA (OR = 0.83; 95% CI = 0.77–0.89) (Table 3).

**TABLE 3:** Pooled coefficients for reporting a QA.^a^

Term	OR	95% CI
(Intercept)	0.48	[0.37; 0.62]
Strength of urges to smoke		
None	2.54	[2.11; 3.05]
Light	0.90	[0.78; 1.04]
Medium strong	1.00	Ref.
Strong	1.06	[0.92; 1.23]
Very strong	0.80	[0.61; 1.05]
Extremely strong	1.47	[0.89; 2.43]
Time	0.77	[0.73; 0.81]
Age	0.95	[0.92; 0.98]
Sex		
Male	1.00.	Ref.
Female	1.26	[1.04; 1.52]
Education		
Low	1.00	Ref.
Middle	1.1	[0.91; 1.32]
High	1.42	[1.16; 1.73]
Region		
Rural	1.00	Ref.
Urban	1.02	[0.90; 1.15]
Metropolitan	0.99	[0.85; 1.15]
Net household income^b^	0.83	[0.77; 0.89]
Interaction of sex:education^c^		
Female:middle	0.94	[0.73; 1.22]
Female:high	0.67	[0.51; 0.89]

Abbreviation: QA, quit attempt.

^a^ At least one quit attempt vs. no quit attempt

^b^ Net household income was standardised into a value range of 0 = lowest to 7 = highest, according to an equalisation technique of the Organisation for Economic Cooperation and Development [27].

^c^ Evaluation of the interaction effect is omitted in the table but explicitly shown in Figure 3.

**FIGURE 3:**
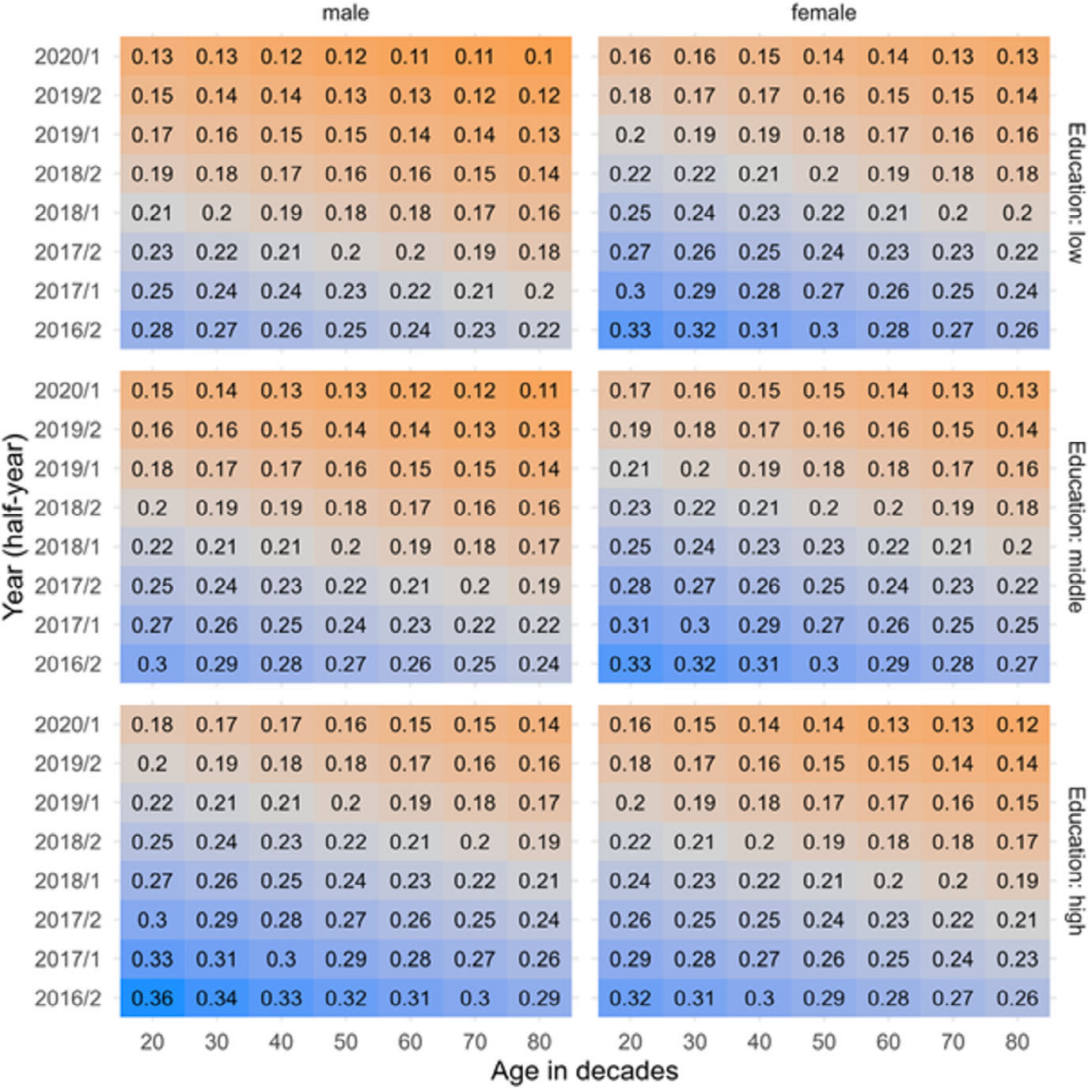
Probabilities of reporting at least one past‐year quit attempt in males and females between 639 February 2016 and January 2020: interaction of participants age (x‐axis) and education (y‐axis).

As outlined in Figure 3, the highest probabilities of reporting a past‐year QA were seen in females between 20 and 40 years and across all educational levels. Similar probabilities in males were observed only among those with high education. No other variables of our data set formed a relevant intersection with regard to QA.

The predictive accuracy of the results was reduced by 3.1% for MTSS and 3.4% for QA when comparing learning with validation data.

## DISCUSSION

The present study consists of a thorough investigation regarding the nature of intersection of co‐occurring demographic and socio‐economic factors for two main predictors for smoking cessation: MTSS and QA. The study yielded three principal findings. First, for both outcomes, the optimal regression model comprised one interaction term.

Two episodes of age were identified with a higher proportion of smokers reporting a MTSS. Higher probabilities were observed in participants between 30 and 50 years from lower and middle (30–40 years) income groups. Those insights are worthwhile in the context of more targeted societal and individualized prevention programs. It is possible that the MTSS is pushed by lower financial resources in middle agers with lower or middle net household income because of several financial requirements at this stage of life (e.g. starting a career and/or a family, educational support for children). Mainly people from lower socio‐economic groups report behavioural steps toward stop smoking in response to increased taxes on tobacco products [53, 54]. This finding could also be used in the adaptation of behavioural smoking cessation programs by combining these challenges of life with health‐related aspects to foster the MTSS. Regardless of educational level, probabilities of reporting at least one past‐year QA were highest in females between 20 and 40 years. Similar probabilities in males were seen only among those with high education. This finding extends the already existing debate on sex differences in smoking cessation. Although women and men do not seem to differ in terms of the likelihood of desire to quit, or the number of quit attempts [55], women seem to be less successful in quitting smoking compared to men [55], and they experience more difficulties maintaining long‐term abstinence than their male counterparts [56]. Quitting among female smokers may be different from quitting among male smokers, also because of life transitions such as becoming pregnant. There is a need for longitudinal studies to determine how consumption patterns (e.g. combustible cigarettes, other tobacco and nicotine products), contextual factors (e.g. smoking status of the partner, voluntary smoking bans at home) or psychosocial factors (e.g. stress, wellbeing) are associated with quitting in females and males. Identifying relevant aspects in the prediction of QA may facilitate targeted smoking cessation programs that adapt treatments by considering both gender and education.

Second, advanced statistical methods like resampling techniques were used to ensure the robustness of the results [18]. We have learned from a recent simulation study that, for example, BE according to the BIC combined with resampling, reduces false‐positive results and reliably identifies relevant differences between intersections [29]. The mechanism that protects against overfitting and improves the generalisability of results is twofold. On the one hand, resampling and training the models on thousands of replicates of the data avoids a regression model being optimal for only one specific data set. On the other hand, predictive accuracy of all regression models is evaluated in data not being used for model training. The combination of both results in a model with the highest possible complexity that is optimal on average over several thousand replications and variants of the data. This model may or may not contain interaction terms. In the latter case, it is not suggestive that intersections with true differences with regard to the outcome are present.

Third, functional forms for continuous covariates such as splines were used to avoid arbitrary categorisation of continuous covariates. To date, heuristic approaches of multi‐variable stratification to form intersections of discrete and categorised continuous covariates are widely used [11, 57]. Adopting this approach to the present study, the stratification variable would comprise 162 levels (2 × 3 × 3 × 3 × 3] [sex (female, male), age (18–39, 40–49 and >50), educational level (low, middle and high), monthly net household income per person (low, middle and high) and region of residence (rural, urban and metropolitan)]. This approach appears to be pragmatic, but it has two major limitations. First, in real‐world data it is impossible to distinguish true from spurious differences between intersections [58]. In simulated data, however, it has been shown [29] that multi‐variable stratification resulted in spurious findings equal to or higher than the number of intersections with true differences. Second, the approach categorises continuous covariates, which is against acknowledged recommendations [28, 30], and, with regard to intersectionality, further inflates spurious findings [14]. Nevertheless, omitting the consideration of interactions between covariates at all, which is common in regression analysis, may not represent the optimal alternative as it could also result in bias [59].

### Strength and limitations

The methods applied in this study represent standards for the development of prediction models [18]. When applied to our intersectionality research questions, it yielded sparse and robust models, whereas the prediction accuracy in data not used for model training was only slightly reduced (3.1% for MTSS, 3.4% for QA). As demonstrated in a recent simulation study [29], the approach is particularly reliable in larger data collections (*n* > 2000). Nevertheless, alternative frameworks that are capable of modelling non‐linear relationships while penalising complexity, such as generalized additive models [17, 60], are also appropriate for examining these research questions. The DEBRA study obtains a large representative data corpus, including repeated survey waves over a period of 4 years and highly standardised data acquisition processes. From this data collection, 9253 past year smokers (current smokers: 8944) contributed to the training data and 3992 (3840) to the validation data, which represents another strength of this study. A study protocol was registered before conducting the study, which enhances transparency, particularly in relation to required modifications, as it is difficult to anticipate all data peculiarities.

Despite these strengths, some limitations should be noted. First, all data were based on self‐report and, therefore, sensitive to recall bias. Second, there is no information about the survey response. Therefore, we were not able to adjust for potential wave‐specifically non‐response. Third, the survey sample is limited to German‐speaking individuals, which reduces the opportunity for migrant participation. This selection may result in lower generalisability of the findings to the whole population in Germany. Fourth, the approach of this study to identify relevant intersections is different from those describing all possible intersections [11, 57], which prevents a comparison of the results. Nevertheless, the final likelihood ratio test showed the relevance of the interaction terms for model enhancement, thereby supporting the existence of pertinent intersections. Overall, however, the predictive performance of the covariates considered is low (i.e. it seems more probable that other characteristics) such as marital status, desire to have children or health events, have a more pronounced influence on both outcomes. Fifth, collapsing categories of the MTSS scale is only partially methodologically justified and represents a clear constraint of this study. This decision was made because of the violation of the proportional odds assumption and to avoid the construction of an overly complex multinomial model. However, this approach is not entirely methodologically sound, because there is only limited methodological guidance on the fusion of categories except for one penalized approach [61].

In sum, the identification of subgroups among currents smokers, which might be overlooked when focusing only on single demographic (e.g. age, sex) or socio‐economic dimensions (e.g. education, income) could contribute to generate new ideas to address them by societal and individualized prevention programs. There is a need for consented recommendations for exploring data to identify relevant instead of all intersections and to avoid non‐reproducible and spurious findings. The approach used in this study can serve as foundation for future studies exploring intersectionality in other addictive behaviours (e.g. alcohol use) to achieve long‐term a reduction of disparities in consumption patterns and to foster health equity.

## AUTHOR CONTRIBUTIONS

**Sabina Ulbricht:** Conceptualization (equal); formal analysis (supporting); methodology (supporting); writing—original draft (lead); writing—review and editing (equal). **Adrian Richter:** Conceptualization (equal); formal analysis (lead); methodology (lead); writing—original draft (equal); writing—review and editing (lead). **Daniel Kotz:** Conceptualization (equal); funding acquisition (lead); methodology (supporting); writing—original draft (supporting); writing—review and editing (supporting). **Sabrina Kastaun:** Conceptualization (equal); methodology (supporting); supervision (lead); writing—original draft (supporting); writing—review and editing (supporting).

## DECLARATION OF INTERESTS

None.

## CLINICAL TRIAL REGISTRATION

German Clinical Trials Register (numbers DRKS00011322, DRKS00017157 and DRKS00028054).

## Supporting information


**Figure S1:** Distribution of age across all DEBRA waves.
**Figure S2:** Distribution of highest educational degree across all DEBRA waves.
**Figure S3:** Proportion of sex across all DEBRA waves.
**Figure S4:** Proportions of participants' residence across all DEBRA waves.
**Figure S5:** Distribution of net household income across all DEBRA waves.
**Figure S6:** Loess‐smoothed prevalence of smokers across all DEBRA waves.
**Figure S7:** Loess‐smoothed prevalence of attempts to quit smoking across all waves of DEBRA.
**Figure S8:** Distribution of all categories of MTSS.
**Figure S9:** Estimated marginal means for the probability of missing heaviness of smoking index.
**Table S1:** Results of the Brant‐test (1) for proportional odds‐assumption*.

## Data Availability

The data underlying this study are third‐party data and are available to researchers on reasonable request. All proposals requesting data access will need to specify how it is planned to use the data, and all proposals will need approval of the DEBRA study team before the data release.The R code used in this study is publicly available in an open GitLab repository. [https://gitlab.com/Adrian_HGW/debra_intersectionality].
