# Supplementary material for: Identifying relevant intersections in relation to motivation and attempt to stop smoking by using a combination of methods to develop robust predictive models and resampling techniques: A cross‐sectional study of the German population
Source: Addiction. 2025 Mar 21;120(9):1863–75. doi: 10.1111/add.70045 (PMC12319644; doi:10.1111/add.70045)
Supplement: Supplementary file 1 — Figure S1: Distribution of age across all DEBRA waves. Figure S2: Distribution of highest educational degree across all DEBRA waves. Figure S3: Proportion of sex across all DEBRA waves. Figure S4: Proportions of participants' residence across all DEBRA waves. Figure S5: Distribution of net household income across all DEBRA waves. Figure S6: Loess‐smoothed prevalence of smokers across all DEBRA waves. Figure S7: Loess‐smoothed prevalence of attempts to quit smoking across all waves of DEBRA. Figure S8: Distribution of all categories of MTSS. Figure S9: Estimated marginal means for the probability of missing heaviness of smoking index. Table S1: Results of the Brant‐test (1) for proportional odds‐assumption*. [file ADD-120-1863-s001.docx]

Supplementary file to

Identifying relevant intersections in relation to motivation and attempt to stop smoking: a cross-sectional study of the German population

Sabina Ulbricht^1^, Adrian Richter^1, 2^, Daniel Kotz^3,4^, Sabrina Kastaun^3,5^

^1^ Department SHIP-KEF, Institute for Community Medicine, University Medicine Greifswald, Greifswald, Germany

^2^ Epidemiology and Health Services Research, German Rheumatology Research Centre Berlin, Berlin, Germany

^3^ Institute of General Practice, Addiction Research and Clinical Epidemiology Unit, Centre for Health and Society, Medical Faculty and University Hospital Düsseldorf, Heinrich Heine University Düsseldorf, Germany

^4^ Department of Behavioural Science and Health, University College London, London, United Kingdom

^5^ Institute of General Practice, Patient-Physician Communication Research Unit, Centre for Health and Society, Medical Faculty and University Hospital Düsseldorf, Heinrich Heine University Düsseldorf, Germany

Content:

[Participant characteristics across DEBRA waves 2](#_Toc179881601)

[Age 2](#_Toc179881602)

[Education 2](#_Toc179881603)

[Sex 3](#_Toc179881604)

[Region of participants residence 3](#_Toc179881605)

[Net household income 4](#_Toc179881606)

[Prevalence of smokers 4](#_Toc179881607)

[Attempts to quit smoking 5](#_Toc179881608)

[Distribution of motivation to stop smoking (MTSS) 5](#_Toc179881609)

[Example of selective missingness 7](#_Toc179881610)

[LR-Tests: MTSS 8](#_Toc179881611)

[LR-Tests: Attempts to quit smoking (ATT) 10](#_Toc179881612)

Supplementary figures:

[Supplementary Figure SF 1: Distribution of age across all DEBRA waves. 2](#_Toc160526089)

[Supplementary Figure SF 2: Distribution of highest educational degree across all DEBRA waves. 2](#_Toc160526090)

[Supplementary Figure SF 3: Proportion of sex across all DEBRA waves. 3](#_Toc160526091)

[Supplementary Figure SF 4: Proportions of participants’ residence across all DEBRA waves. 3](#_Toc160526092)

[Supplementary Figure SF 5: Distribution of net household income across all DEBRA waves. 4](#_Toc160526093)

[Supplementary Figure SF 6: Loess-smoothed prevalence of smokers across all DEBRA waves. 4](#_Toc160526094)

[Supplementary Figure SF 7: Loess-smoothed prevalence of attempts to quit smoking across all waves of DEBRA. 5](#_Toc160526095)

[Supplementary Figure SF 8: Distribution of all categories of MTSS. 5](#_Toc160526096)

[Supplementary Figure SF 9: Estimated marginal means for the probability of missing heaviness of smoking index. 7](#_Toc160526097)

Supplementary tables:

[Supplementary Table ST 1: Results of the Brant-test (1) for proportional odds-assumption. 6](#_Toc160526273)

# Participant characteristics across DEBRA waves

## Age


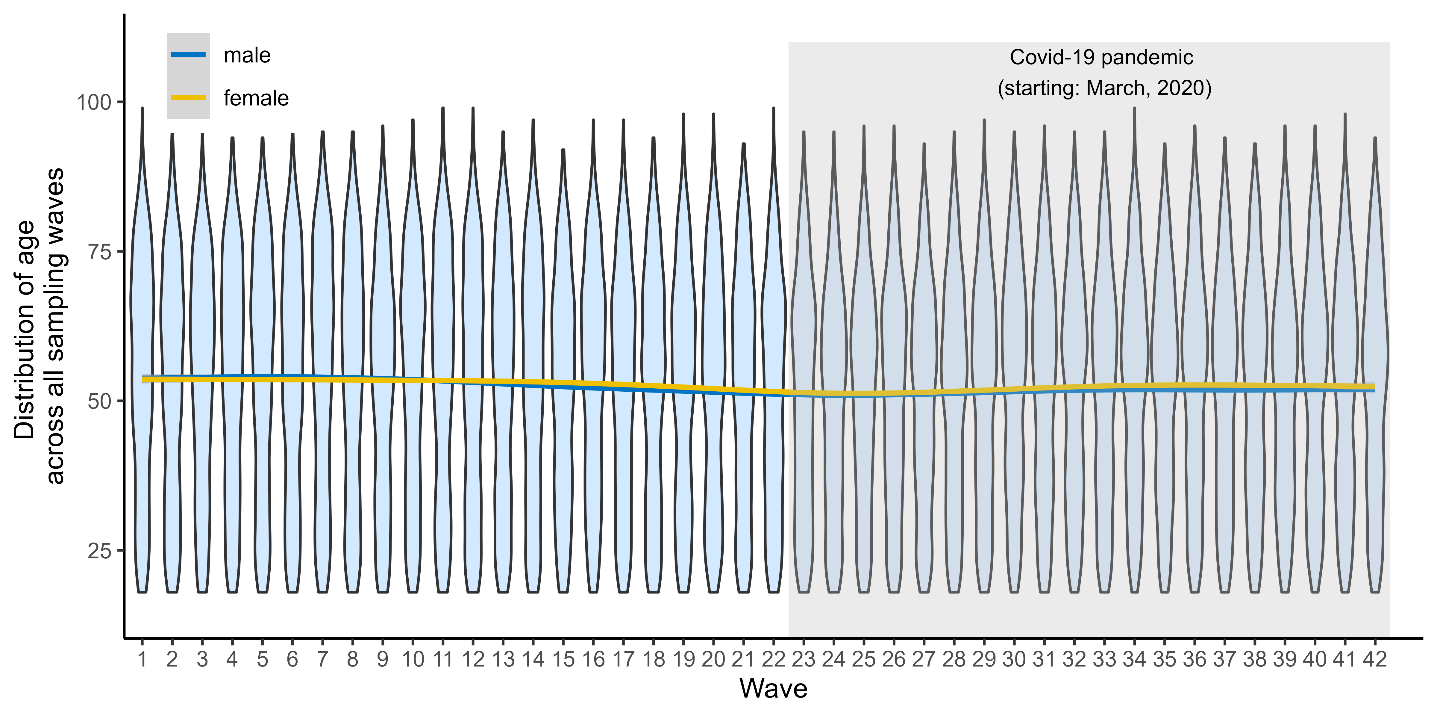


Supplementary Figure SF 1: Distribution of age across all DEBRA waves.

## Education


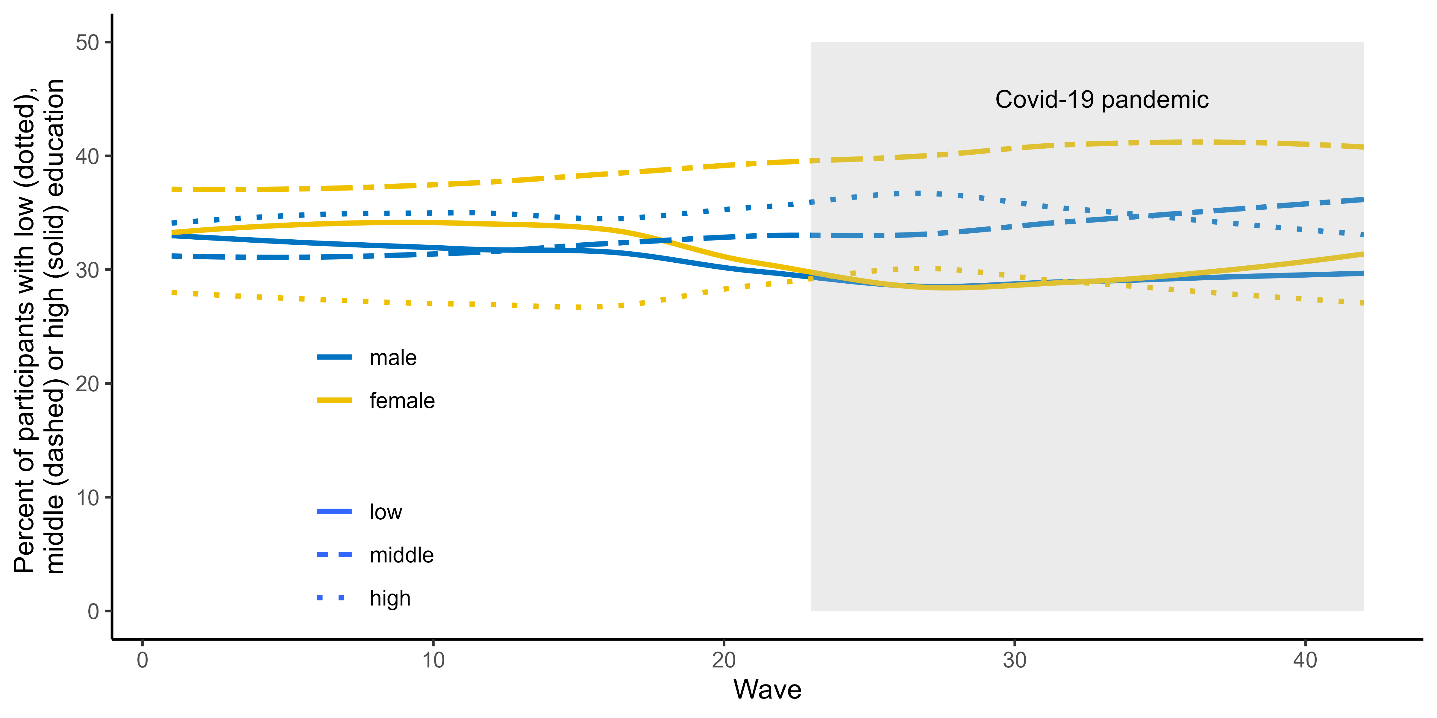


Supplementary Figure SF 2: Distribution of highest educational degree across all DEBRA waves.

## Sex


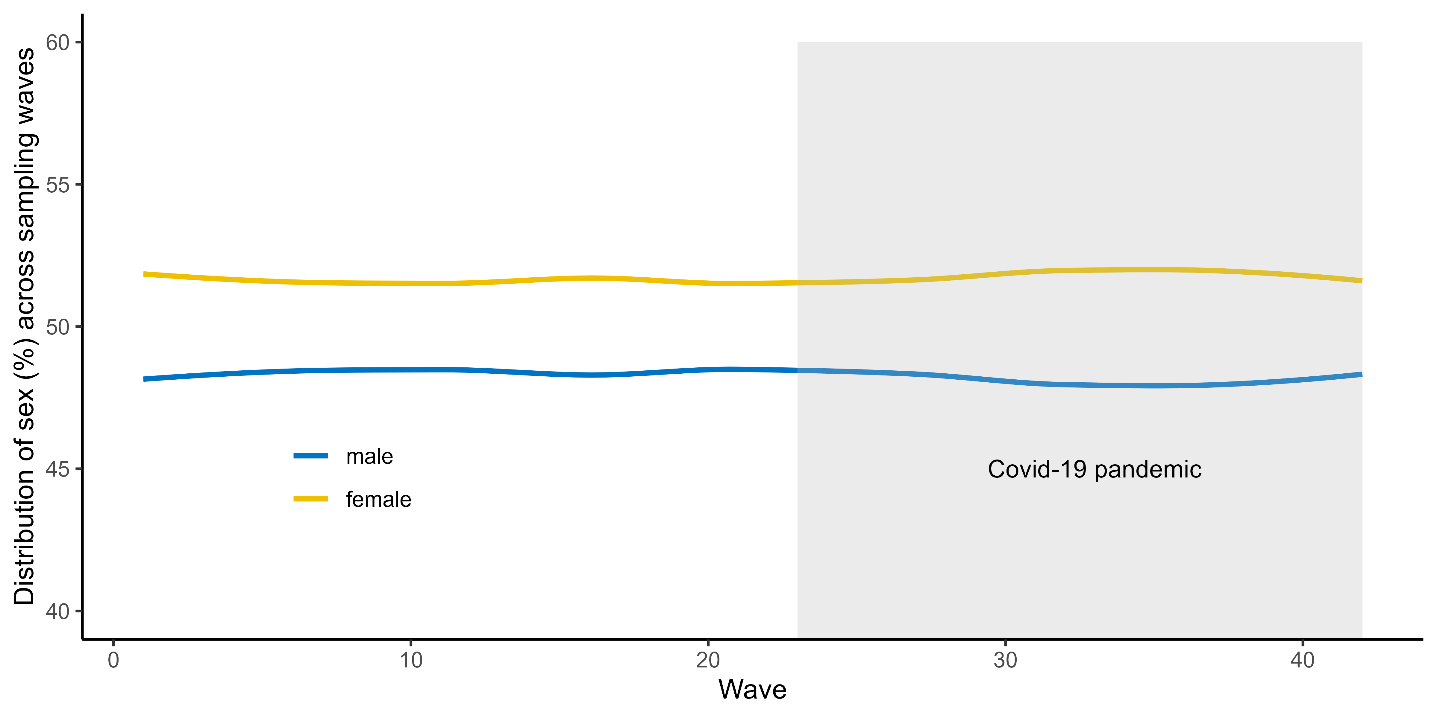


Supplementary Figure SF 3: Proportion of sex across all DEBRA waves.

## Region of participants residence


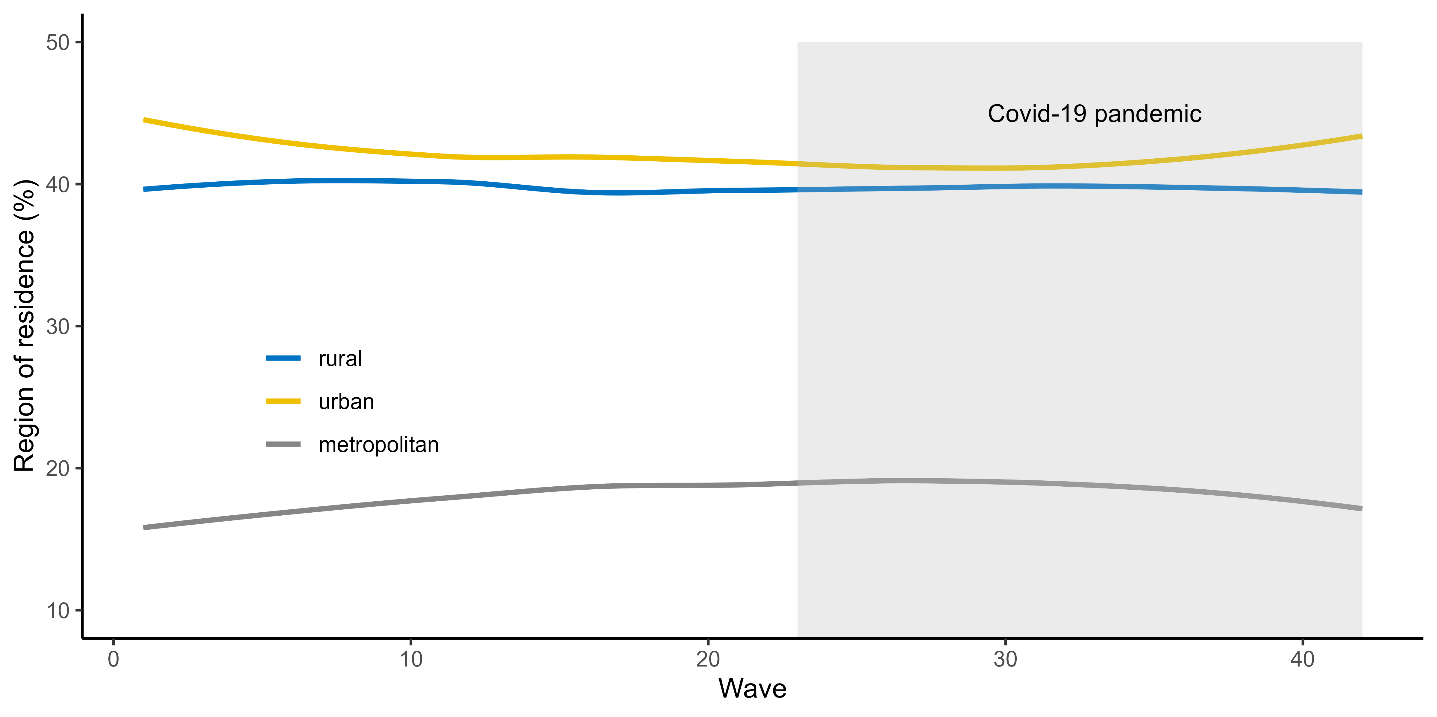


Supplementary Figure SF 4: Proportions of participants’ residence across all DEBRA waves.

## Net household income


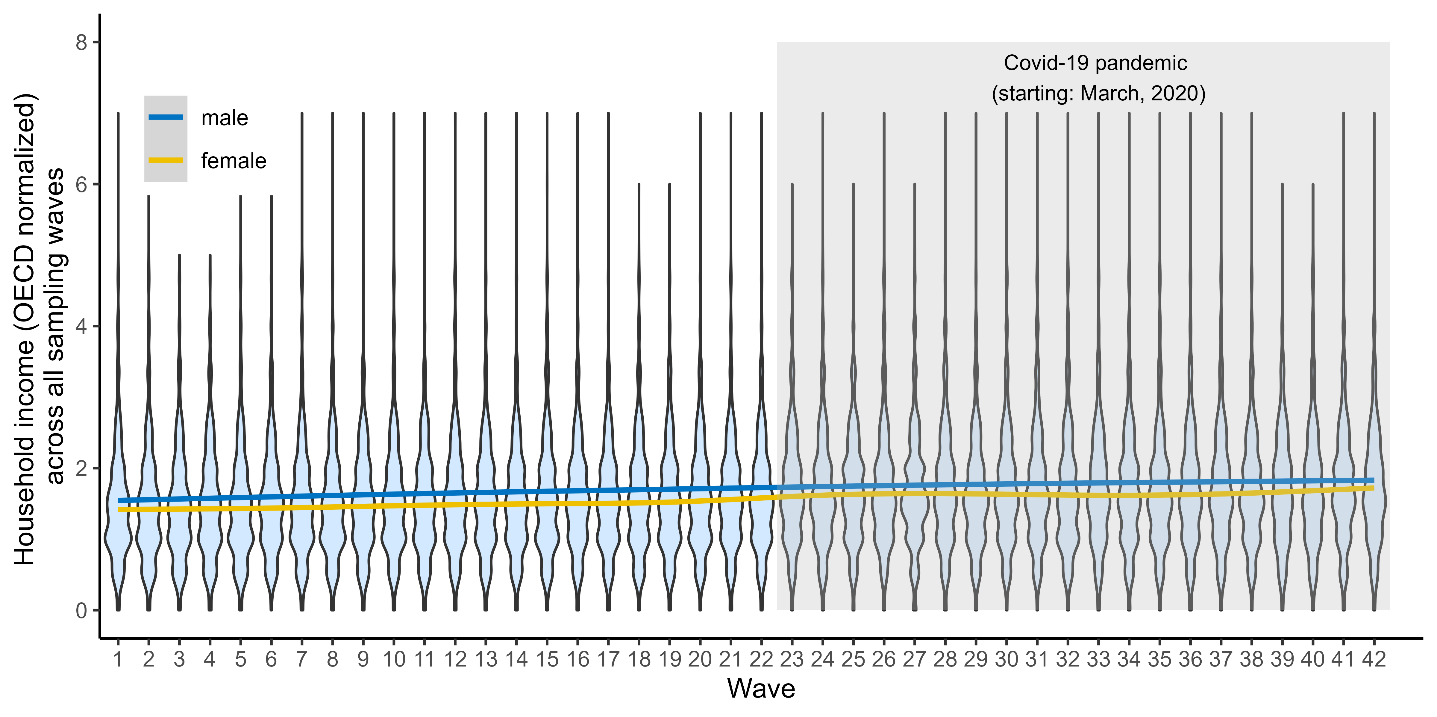


Supplementary Figure SF 5: Distribution of net household income across all DEBRA waves.

## Prevalence of smokers


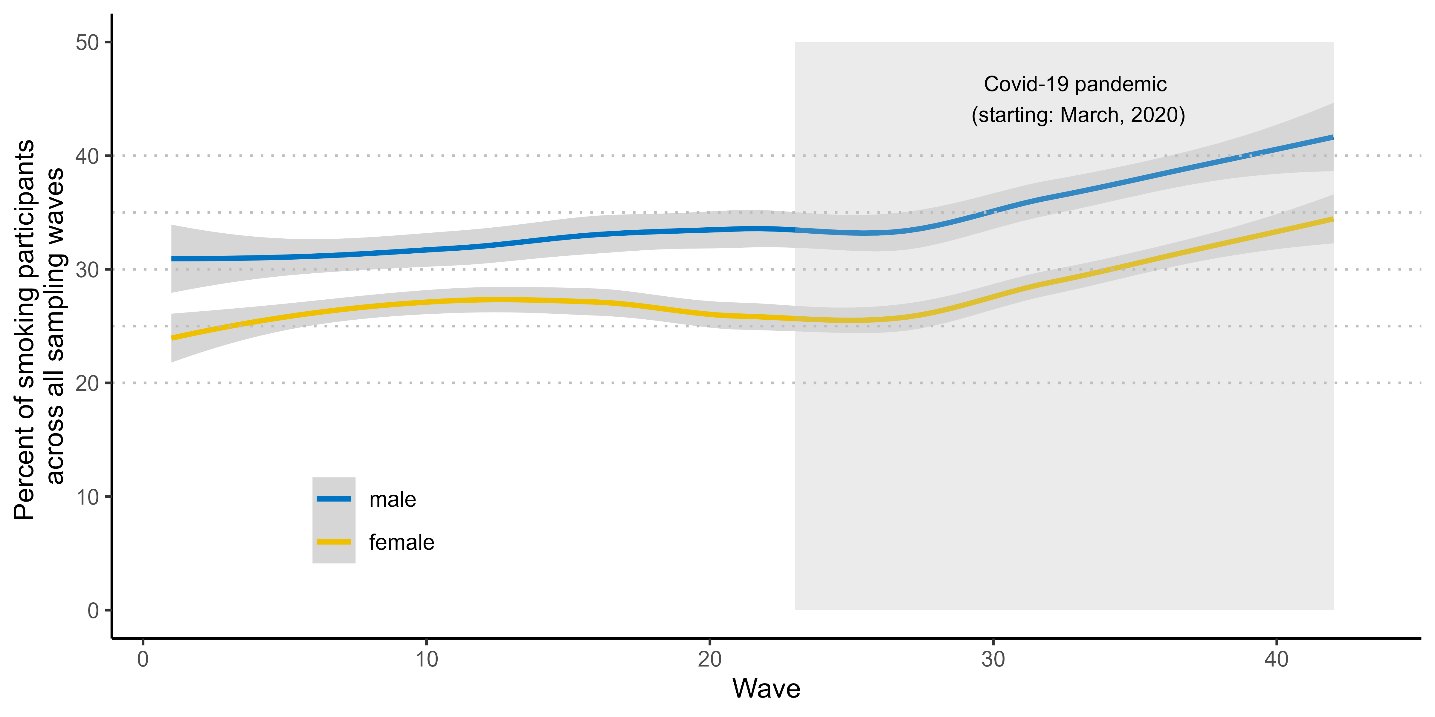


Supplementary Figure SF 6: Loess-smoothed prevalence of smokers across all DEBRA waves.

## Attempts to quit smoking


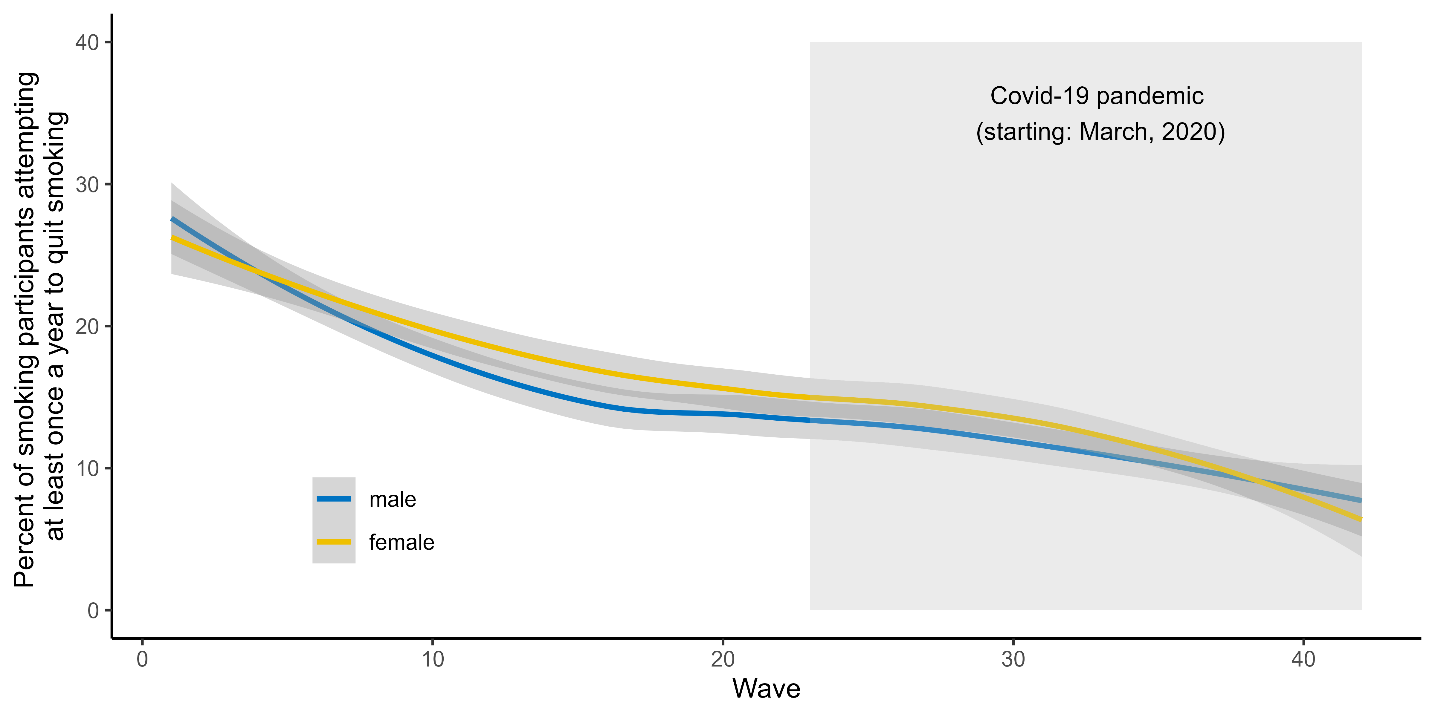


Supplementary Figure SF 7: Loess-smoothed prevalence of attempts to quit smoking across all waves of DEBRA.

# Distribution of motivation to stop smoking (MTSS)


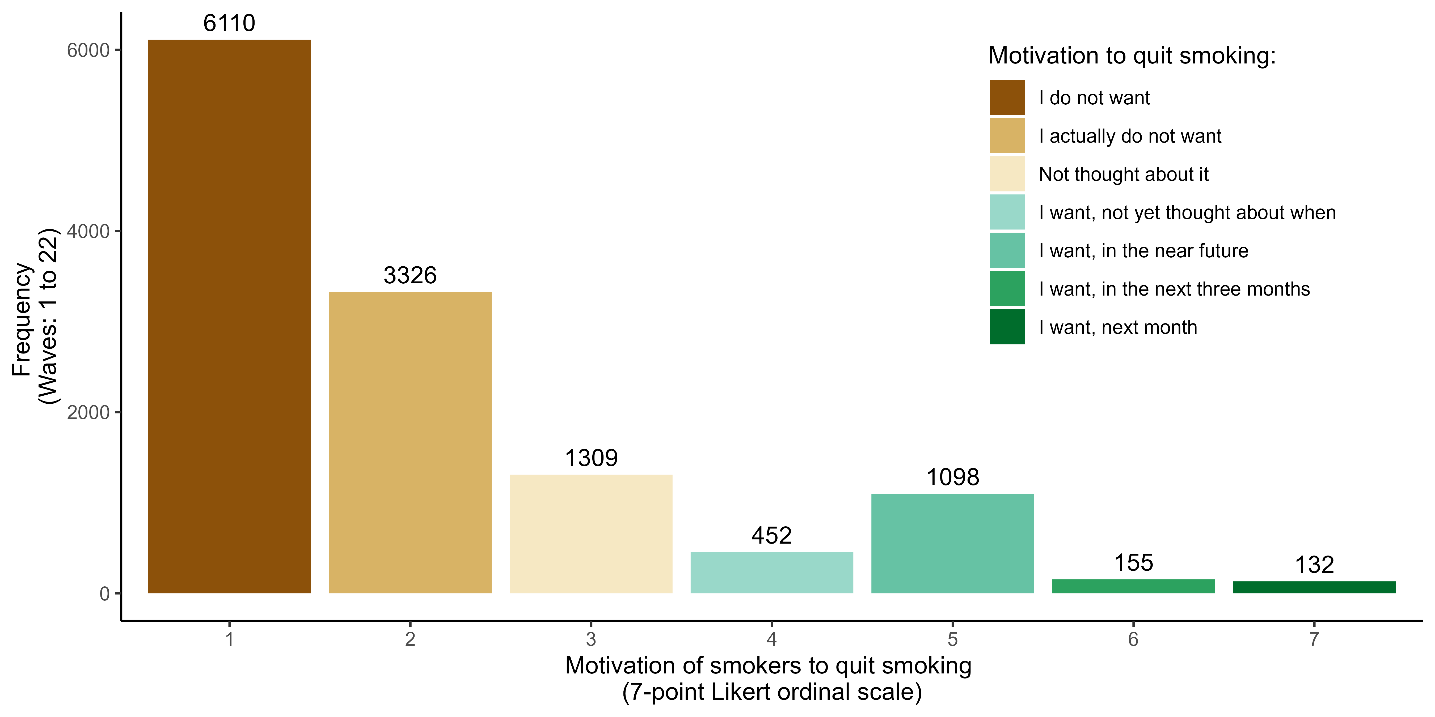


Supplementary Figure SF 8: Distribution of all categories of MTSS.

Supplementary Table ST 1: Results of the Brant-test (1) for proportional odds-assumption*.

| **Effect** | **b[polr]** | **b[>1]** | **b[>2]** | **ChiSqrt** | **P** |
| --- | --- | --- | --- | --- | --- |
| hsi | -0.075 | -0.072 | -0.084 | 0.48 | 0.489 |
| time | -0.096 | -0.067 | -0.188 | 24.08 | 0 |
| alter | -0.005 | -0.004 | -0.006 | 0.86 | 0.352 |
| sexfemale | 0.085 | 0.094 | 0.054 | 0.59 | 0.441 |
| educationmiddle | 0.163 | 0.171 | 0.124 | 0.58 | 0.447 |
| educationhigh | 0.329 | 0.334 | 0.309 | 0.12 | 0.729 |
| incomeOECD | -0.049 | -0.034 | -0.097 | 3.32 | 0.069 |
| region2provincial | 0.140 | 0.153 | 0.108 | 0.59 | 0.443 |
| region2municipal | -0.054 | -0.057 | -0.037 | 0.08 | 0.783 |

* After collapsing unspecific willingness to stop smoking (MTSS = 2 or 3) and more definite willingness (MTSS = 4 - 7) still deviations from proportional odds are found. The hypothesis underlying the Brant test is “H0: the proportional odds assumption is correct” which must be rejected for covariate time. The latter are substantially higher if less categories are collapsed, i.e. adhering more closely to the original measurement scale introduces more often violations of the proportional odds assumption. The Brant-test is implemented in the R package car (2).

# Example of selective missingness

The missingness in heaviness of smoking index (HSI) was overall <10%. The following figure shows probabilities of a missing HSI that were estimated using multiple logistic regression and adjusted for age, sex, and education. The binary outcome was defined as 1=HIS missing, 0=HIS reported.


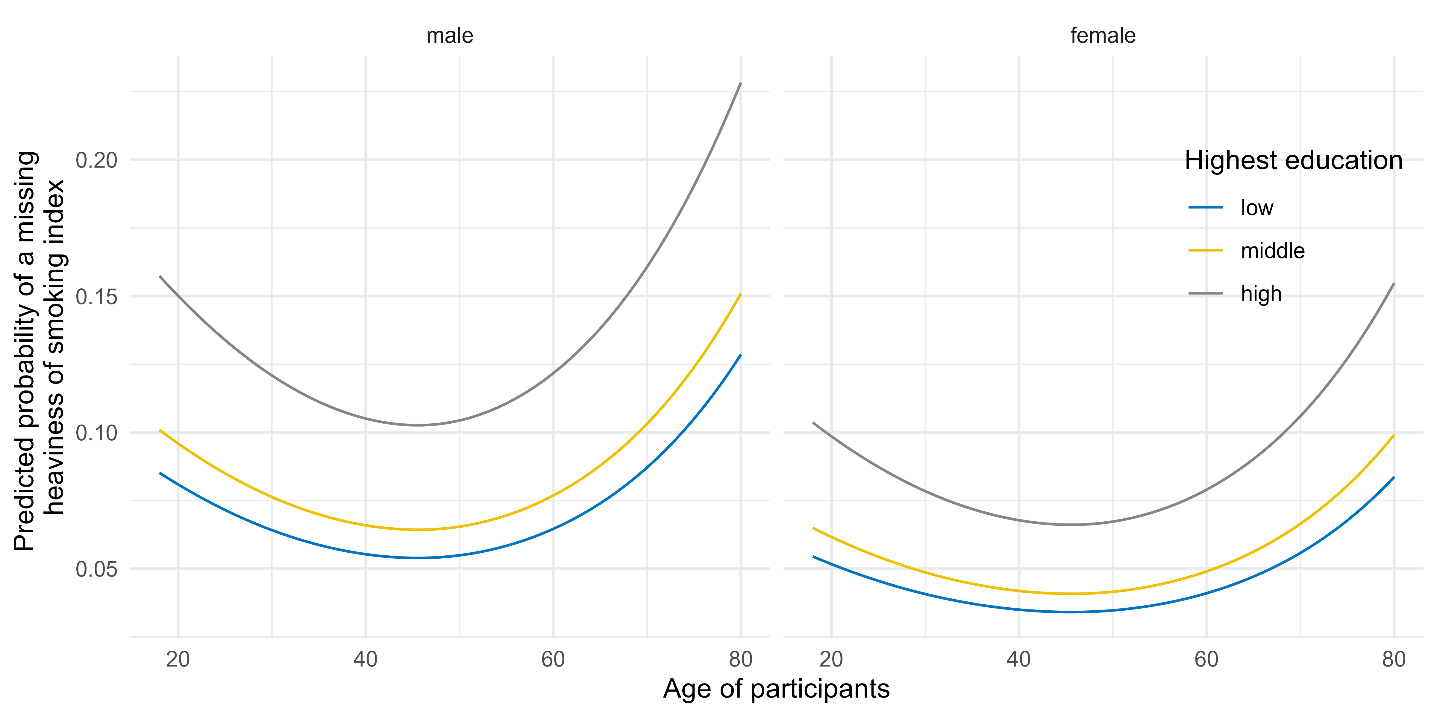


Supplementary Figure SF 9: Estimated marginal means for the probability of missing heaviness of smoking index.

# LR-Tests: MTSS

Likelihood ratio test over all imputations comparing the final model including interaction term vs the simpler model without interaction term.

$`1`

Likelihood ratio test

Model 1: mtss ~ sturge + ns(time, df = 3) + ns(alter, df = 4) + sex +

education + region2 + ns(incomeOECD, df = 2)

Model 2: mtss ~ sturge + ns(time, df = 3) + ns(alter, df = 4) + sex +

education + region2 + ns(incomeOECD, df = 2) + ns(alter,

df = 4):ns(incomeOECD, df = 2)

#Df LogLik Df Chisq Pr(>Chisq)

1 40 -8800.7

2 56 -8784.6 16 32.299 0.009141 **

---

$`2`

Likelihood ratio test

Model 1: mtss ~ sturge + ns(time, df = 3) + ns(alter, df = 4) + sex +

education + region2 + ns(incomeOECD, df = 2)

Model 2: mtss ~ sturge + ns(time, df = 3) + ns(alter, df = 4) + sex +

education + region2 + ns(incomeOECD, df = 2) + ns(alter,

df = 4):ns(incomeOECD, df = 2)

#Df LogLik Df Chisq Pr(>Chisq)

1 40 -8806.6

2 56 -8790.2 16 32.768 0.007932 **

---

$`3`

Likelihood ratio test

Model 1: mtss ~ sturge + ns(time, df = 3) + ns(alter, df = 4) + sex +

education + region2 + ns(incomeOECD, df = 2)

Model 2: mtss ~ sturge + ns(time, df = 3) + ns(alter, df = 4) + sex +

education + region2 + ns(incomeOECD, df = 2) + ns(alter,

df = 4):ns(incomeOECD, df = 2)

#Df LogLik Df Chisq Pr(>Chisq)

1 40 -8798.9

2 56 -8783.6 16 30.601 0.01512 *

---

$`4`

Likelihood ratio test

Model 1: mtss ~ sturge + ns(time, df = 3) + ns(alter, df = 4) + sex +

education + region2 + ns(incomeOECD, df = 2)

Model 2: mtss ~ sturge + ns(time, df = 3) + ns(alter, df = 4) + sex +

education + region2 + ns(incomeOECD, df = 2) + ns(alter,

df = 4):ns(incomeOECD, df = 2)

#Df LogLik Df Chisq Pr(>Chisq)

1 40 -8813.9

2 56 -8796.9 16 33.995 0.005442 **

---

$`5`

Likelihood ratio test

Model 1: mtss ~ sturge + ns(time, df = 3) + ns(alter, df = 4) + sex +

education + region2 + ns(incomeOECD, df = 2)

Model 2: mtss ~ sturge + ns(time, df = 3) + ns(alter, df = 4) + sex +

education + region2 + ns(incomeOECD, df = 2) + ns(alter,

df = 4):ns(incomeOECD, df = 2)

#Df LogLik Df Chisq Pr(>Chisq)

1 40 -8802.4

2 56 -8786.0 16 32.843 0.007752 **

---

$`6`

Likelihood ratio test

Model 1: mtss ~ sturge + ns(time, df = 3) + ns(alter, df = 4) + sex +

education + region2 + ns(incomeOECD, df = 2)

Model 2: mtss ~ sturge + ns(time, df = 3) + ns(alter, df = 4) + sex +

education + region2 + ns(incomeOECD, df = 2) + ns(alter,

df = 4):ns(incomeOECD, df = 2)

#Df LogLik Df Chisq Pr(>Chisq)

1 40 -8801.8

2 56 -8785.9 16 31.857 0.01044 *

---

$`7`

Likelihood ratio test

Model 1: mtss ~ sturge + ns(time, df = 3) + ns(alter, df = 4) + sex +

education + region2 + ns(incomeOECD, df = 2)

Model 2: mtss ~ sturge + ns(time, df = 3) + ns(alter, df = 4) + sex +

education + region2 + ns(incomeOECD, df = 2) + ns(alter,

df = 4):ns(incomeOECD, df = 2)

#Df LogLik Df Chisq Pr(>Chisq)

1 40 -8804.5

2 56 -8788.9 16 31.28 0.01239 *

---

$`8`

Likelihood ratio test

Model 1: mtss ~ sturge + ns(time, df = 3) + ns(alter, df = 4) + sex +

education + region2 + ns(incomeOECD, df = 2)

Model 2: mtss ~ sturge + ns(time, df = 3) + ns(alter, df = 4) + sex +

education + region2 + ns(incomeOECD, df = 2) + ns(alter,

df = 4):ns(incomeOECD, df = 2)

#Df LogLik Df Chisq Pr(>Chisq)

1 40 -8800.4

2 56 -8784.8 16 31.215 0.01263 *

---

$`9`

Likelihood ratio test

Model 1: mtss ~ sturge + ns(time, df = 3) + ns(alter, df = 4) + sex +

education + region2 + ns(incomeOECD, df = 2)

Model 2: mtss ~ sturge + ns(time, df = 3) + ns(alter, df = 4) + sex +

education + region2 + ns(incomeOECD, df = 2) + ns(alter,

df = 4):ns(incomeOECD, df = 2)

#Df LogLik Df Chisq Pr(>Chisq)

1 40 -8811.1

2 56 -8794.1 16 33.912 0.005584 **

---

$`10`

Likelihood ratio test

Model 1: mtss ~ sturge + ns(time, df = 3) + ns(alter, df = 4) + sex +

education + region2 + ns(incomeOECD, df = 2)

Model 2: mtss ~ sturge + ns(time, df = 3) + ns(alter, df = 4) + sex +

education + region2 + ns(incomeOECD, df = 2) + ns(alter,

df = 4):ns(incomeOECD, df = 2)

#Df LogLik Df Chisq Pr(>Chisq)

1 40 -8799.6

2 56 -8783.3 16 32.606 0.008332 **

# LR-Tests: Attempts to quit smoking (ATT)

Likelihood ratio test over all imputations (10) comparing the final model including interaction term vs the simpler model without interaction term.

$`1`

Likelihood ratio test

Model 1: att ~ sturge + time + I(alter/10) + sex + region2 + incomeOECD +

education

Model 2: att ~ sturge + time + I(alter/10) + sex + region2 + incomeOECD +

education + sex:education

#Df LogLik Df Chisq Pr(>Chisq)

1 14 -4371.4

2 16 -4366.4 2 10.142 0.006277 **

---

$`2`

Likelihood ratio test

Model 1: att ~ sturge + time + I(alter/10) + sex + region2 + incomeOECD +

education

Model 2: att ~ sturge + time + I(alter/10) + sex + region2 + incomeOECD +

education + sex:education

#Df LogLik Df Chisq Pr(>Chisq)

1 14 -4352.0

2 16 -4347.4 2 9.1197 0.01046 *

---

$`3`

Likelihood ratio test

Model 1: att ~ sturge + time + I(alter/10) + sex + region2 + incomeOECD +

education

Model 2: att ~ sturge + time + I(alter/10) + sex + region2 + incomeOECD +

education + sex:education

#Df LogLik Df Chisq Pr(>Chisq)

1 14 -4354.8

2 16 -4350.5 2 8.5246 0.01409 *

---

$`4`

Likelihood ratio test

Model 1: att ~ sturge + time + I(alter/10) + sex + region2 + incomeOECD +

education

Model 2: att ~ sturge + time + I(alter/10) + sex + region2 + incomeOECD +

education + sex:education

#Df LogLik Df Chisq Pr(>Chisq)

1 14 -4386.8

2 16 -4382.3 2 9.0909 0.01062 *

---

$`5`

Likelihood ratio test

Model 1: att ~ sturge + time + I(alter/10) + sex + region2 + incomeOECD +

education

Model 2: att ~ sturge + time + I(alter/10) + sex + region2 + incomeOECD +

education + sex:education

#Df LogLik Df Chisq Pr(>Chisq)

1 14 -4371.3

2 16 -4367.0 2 8.637 0.01332 *

---

$`6`

Likelihood ratio test

Model 1: att ~ sturge + time + I(alter/10) + sex + region2 + incomeOECD +

education

Model 2: att ~ sturge + time + I(alter/10) + sex + region2 + incomeOECD +

education + sex:education

#Df LogLik Df Chisq Pr(>Chisq)

1 14 -4361.4

2 16 -4356.2 2 10.464 0.005344 **

---

$`7`

Likelihood ratio test

Model 1: att ~ sturge + time + I(alter/10) + sex + region2 + incomeOECD +

education

Model 2: att ~ sturge + time + I(alter/10) + sex + region2 + incomeOECD +

education + sex:education

#Df LogLik Df Chisq Pr(>Chisq)

1 14 -4362.9

2 16 -4358.2 2 9.3888 0.009147 **

---

$`8`

Likelihood ratio test

Model 1: att ~ sturge + time + I(alter/10) + sex + region2 + incomeOECD +

education

Model 2: att ~ sturge + time + I(alter/10) + sex + region2 + incomeOECD +

education + sex:education

#Df LogLik Df Chisq Pr(>Chisq)

1 14 -4375.9

2 16 -4371.5 2 8.8376 0.01205 *

---

$`9`

Likelihood ratio test

Model 1: att ~ sturge + time + I(alter/10) + sex + region2 + incomeOECD +

education

Model 2: att ~ sturge + time + I(alter/10) + sex + region2 + incomeOECD +

education + sex:education

#Df LogLik Df Chisq Pr(>Chisq)

1 14 -4360.6

2 16 -4356.2 2 8.7022 0.01289 *

---

$`10`

Likelihood ratio test

Model 1: att ~ sturge + time + I(alter/10) + sex + region2 + incomeOECD +

education

Model 2: att ~ sturge + time + I(alter/10) + sex + region2 + incomeOECD +

education + sex:education

#Df LogLik Df Chisq Pr(>Chisq)

1 14 -4371.7

2 16 -4367.8 2 7.6739 0.02156 *

---
